# MicroRNA-338 Inhibits Growth, Invasion and Metastasis of Gastric Cancer by Targeting NRP1 Expression

**DOI:** 10.1371/journal.pone.0094422

**Published:** 2014-04-15

**Authors:** Yang Peng, Yan-Min Liu, Lu-Chun Li, Lu-Lu Wang, Xiao-Ling Wu

**Affiliations:** Department of Gastroenterology and Hepatology, the Second Affiliated Hospital of Chongqing Medical University, Chongqing, China; China Medical University, Taiwan

## Abstract

NRP1 as multifunctional non-tyrosine-kinase receptors play critical roles in tumor progression. MicroRNAs (miRNAs) are an important class of pervasive genes that are involved in a variety of biological functions, particularly cancer. It remains unclear whether miRNAs can regulate the expression of NRP1. The goal of this study was to identify miRNAs that could inhibit the growth, invasion and metastasis of gastric cancer by targeting NRP1 expression. We found that miR-338 expression was reduced in gastric cancer cell lines and in gastric cancer tissues. Moreover, we found that miR-338 inhibited gastric cancer cell migration, invasion, proliferation and promoted apoptosis by targeting NRP1 expression. As an upstream regulator of NRP1, miR-338 directly targets NRP1. The forced expression of miR-338 inhibited the phosphorylation of Erk1/2, P38 MAPK and Akt; however, the expression of phosphorylated Erk1/2, P38 MAPK and Akt was restored by the overexpression of NRP1. In AGS cells infected with miR-338 or transfected with SiNRP1, the protein levels of fibronectin, vimentin, N-cadherin and SNAIL were decreased, but the expression of E-cadherin was increased. The expression of mesenchymal markers in miR-338-expressing cells was restored to normal levels by the restoration of NRP1 expression. *In vivo*, miR-338 also decreased tumor growth and suppressed D-MVA by targeting NRP1. Therefore, we conclude that miR-338 acts as a novel tumor suppressor gene in gastric cancer. miR-338 can decrease migratory, invasive, proliferative and apoptotic behaviors, as well as gastric cancer EMT, by attenuating the expression of NRP1.

## Introduction

Neuropilins, including neuropilin1 (NRP1) and neuropilin2 (NRP2), are multifunctional non-tyrosine-kinase receptors that were first identified based on their critical roles in the developing nervous system[1]. Nrp1 and Nrp2 have 44% homology and share many structural and biological properties. NRP1 mainly exists in bloodvesselendothelia, and NRP2 is mainly found in lymphatic vessels [2], [3]. Subsequent investigations identified NRP-1 as a receptor for the vascular endothelial growth factor (VEGF)-A isoform VEGF-165 in both endothelial cells and some tumor cells [4], [5]. Research has shown that NRP1 is up-regulated in multiple tumor types and is expressed in different tumor vasculatures [3], [6], suggesting that NRP1 plays a critical role in tumor progression. Early research revealed that NRP1 could affect the growth of tumors as a coreceptor of VEGFR [5]. Scientists later found that NRP1 could boost tumorangiogenesis, accelerate tumor growth and curb tumor apoptosis without VEGFR [7]. NRP1 can be a coreceptor of other growth factors, which elucidates why VEGF can signal through neuropilins in the absence of VEGFR-1or VEGFR-2[8], [9]. As a coreceptor of TβRI/TβRII, NRP1 can curb tumor apoptosis and accelerate tumor growth via both canonical and non-canonical signaling [10]. NRP1, as a coreceptor of PDGFR/cmet, can boost tumorangiogenesis via P38MAPK and ERK signaling[11].

As upstream regulatory genes of NRP1, the transcription factors sp1/3 and AP1 can regulate the expression of NRP1 [12]. Some research has shown that butyrate suppresses the expression of neuropilin I in colorectal cell lines through the inhibition of Sp1 transactivation [13].

MicroRNAs (miRNAs) are non-coding RNA molecules approximately 21–23 nucleotides in length that regulate gene expression at the post-transcriptional level [14], [15], [16]. miRNA expression profiling analyses have revealed a global down-regulation of mature miRNA levels in primary human tumors relative to normal tissues [17], [18]. Thus, miRNAs may function as tumor suppressors or oncogenes, and dysregulated miRNA expression might contribute to tumor cell metastasis. It remains unclear whether miRNAs can regulate the expression of NRP1; therefore, we aimed to determine the target miRNAs of NRP1. Additional functions of NRP1 may be discovered by studying the target miRNAs of NRP1.

## Materials and Methods

### Human Tissue Specimens and Cell Lines

This study utilized fresh tissues, including 41 human gastric cancer samples and 24 samples of adjacent normal mucosal tissues derived from 41 patients who underwent surgery at the Second Affiliated Hospital of Chongqing Medical University between 2012 and 2013. This study was conducted according to the ‘Biomedical Research Involving Human Ethics Review (Tentative)’ regulation of the Ministry of Health and the Declaration of Helsinki on Ethical Principles for Medical Research Involving Human Subjects. All samples were obtained with the informed consent of the patients and the experiments were approved by the Institutional Review Board of the Second Affiliated Hospital of Chongqing Medical University. All participants provided written informed consent to participate in this study.

The SGC-7901, HGC-27, AGS, MKN-45 and N87 cell lines were obtained from the American Type Culture Collection (ATCC; Manassas, VA, USA), and the GES-1 cell line was purchased from the Type Culture Collection of the Chinese Academy of Sciences(Shanghai, China). The cell lines were cultured in RPMI1640 (Hyclone, Logan Utah USA) supplemented with 10% fetal bovine serum (FBS) and were incubated at 37°C with 5% CO2.

### Primers, RNA Isolation and miRNA Detection

The primers for miR-338-3p and U6 were produced using the miScript Primer Assay kit (Qiagen Dusseldorf Germany). The sequences of the miRNAs used in this study were as follows: miR-338-3p: UCCAGCAUC- AGUGAUUUUGUUG and U6: CGCAAGGAUGACACGCAAAUUCGUGAA- GCGUUCCAUAUUUUU. The reverse primers were also used in the reverse transcription step. Total miRNA was extracted from cultured cells and human tissue specimens using RNAiso for Small RNA (TaKaRa Bio,Otsu, Japan) according to the manufacturer's instructions. Poly-A tails were added to miR-338 and U6 with the miRNA Reaction Buffer Mix (TaKaRa Bio), and then cDNA was synthesized from 5 ng of total RNA using the miRNA PrimeScript RT Enzyme Mix (TaKaRa Bio). Real-time PCR was performed in a CFX96 Real-Time PCR Detection System (Bio-Rad) with SYBR Premix Ex Taq II (TaKaRa Bio). The PCR conditions were 95°C for 30 s, followed by 40 cycles of 95°C for 5 s and 60°C for 30 s. The data were normalized against the U6 snRNA. After amplification, a melting curve analysis was performed to confirm the specificity of the products.

### pcDNA Expression Plasmids and Plasmid Transfection

The ORF sequence of NRP1 was amplified from genomic DNA isolated from the AGS cell line, and the ORF region of the NRP1 cDNA was then subcloned into the GV230 vector (GeneChem Corporation, Shanghai, China).The plasmid was transfected into AGS and MKN45 cells using Lipofectamine 2000 (Invitrogen).Twenty-four hours after transfection, the cells were used for a rescue experiment. AGS cells that were stably transfected with NRP1 were selected using 2 ug/uL puromycin (Invitrogen, Cergy-Pontoise, France) 48 h after the transfection.

### miRNA Gene Cloning and Ectopic Expression

The human miR-338 gene was PCR-amplified from normal genomic DNA and cloned into a lentiviral vector. The lentiviruses were generated by the cotransfection of HEK293T cells with plasmids pGC-LV, pHelper 1.0 and pHelper 2.0 using Lipofectamine 2000 (Invitrogen). Viruses were harvested 48 h post-transfection, and the infections were performed in the presence of 2 mg/mL polybrene (GeneChem Corporation). Control miRNA viruse was purchased from GeneChem Corporation (Shanghai, China). AGS cells that were stably infected with miR-338 were selected using 2 ug/uL puromycin (Invitrogen) 48 h after the infection.

### Gene Expression Knockdown by RNA-Interference

NRP1 expression by AGS cells was silenced by transfecting the following targeted siRNA sequences (Sangon Biotech, Shanghai, China) with Lipofectamine 2000 (Invitrogen); #1: agatcgacgttagctccaa;#2: aacacctagtggagtgata; #3: CAATCACGTGCAGGCTCAA(where not specified, siRNA #3 was used). Control siRNAs (siC) were generated by introducing 4 base substitutions in NRP1 targeting sequence (GATAGGTCATGACTGCCC). Forty-eight hours after transfection, NRP1 expression was examined by immunoblotting.

### Luciferase Reporter Assay

A PsicheckTM-2 Dual-Luciferase miRNA target expression vector was used for 3′UTR luciferase assays (Sangon Biotech, Shanghai, China). The target oncogene of miRNA-338 was selected on the basis of the online microRNA target database http://www.microrna.org/microrna/home.do. The primer sequences for the wild-type 3′UTR were as follows: forward: 5′- CCGCTCGAG CAAAGGAC- GGAAGTGGAAGG -3′ and reverse 5′-ATTTGCGGCCGC GGAGTTCACAAGCACGAGG- TT-3′. Because there are two binding sites in the NRP1 3′UTR, we designed two primer sequences for the mutant 3′UTR. For one mutant 3′UTR, the primer sequences were the following: forward: 5′- GAAT- AATCAGGCATCTTTGTTGAGACCAAGTATGATT-3′ and reverse: 5′- GGTCTCAACAAAG- ATGCCTGATTATTCAAATGAAACC-3′. For the other mutant 3′UTR, the primer sequences were as follows: forward: 5′- GCGAAATCCAAGGCATCTTCACCAAGCGTATTCCGTGT-3′ and reverse: 5′- GCTTGGTGAAGATGCCTTGGATTTCGCTCAGTTTCC-3′. For the luciferase assay, Lipofectamine 2000 was used to cotransfect MKN45 cells with the hsa-miR-338 and PsicheckTM-2 Dual-Luciferase miRNA target expression vectors containing wild-type or mutant target sequences. The firefly luciferase activity was measured using the Dual Luciferase Assay (Promega, Madison, WI, USA) 18 h after transfection, and the results were normalized against Renilla luciferase. Each reporter plasmid was transfected at least three times (on different days), and each sample was assayed in triplicate.

### Cell Viability and Clonability Assays

The transfected cells were seeded into 96-well plates at a density of 1×10^4^ cells/well. An MTT solution (20 ul of 5 mg/ml MTT) was added to the cultures (for a total volume of 200 ul) and incubated for 4 hat 37°C. Following the removal of the culture medium, the remaining crystals were dissolved in DMSO, and the absorbance at 570 nm was measured. For the colony formation assay, cells were seeded at a low density (1000 cells/plate) and allowed to grow until visible colonies appeared. The cells were then stained with Giemsa, and the colonies were counted.

### Migration and Invasion Assays

For the transwell migration assays, 10×10^4^ cells were plated in the top chamber with a non-coated membrane (24-well insert; 8 mm pore size; BD Biosciences). For the invasion assays, 2×10^5^ cells were plated in the top chamber with a Matrigel-coated membrane (24-well insert; 8 mm pore size; BD Biosciences). For both assays, the cells were plated in a serum-free medium, and a medium supplemented with 10% serum was used as a chemoattractant in the lower chamber. The cells were incubated for 16 h at 37°C and 5% CO2 in a tissue culture incubator. After 16 h, the non-migrated/non-invading cells were removed from the upper sides of the transwell membrane filter inserts using cotton-tipped swabs. The migrated/invaded cells on the lower sides of the inserts were stained with Giemsa, and the cells were counted.

### Antibodies

Antibodies against fibronectin, vimentin, N-cadherin, E-cadherin, SNAIL and GAPDH were purchased from Santa Cruz Biotechnology (CA, USA). Phospho-Erk1/2, phospho-Akt, and phospho-P38MAPK were purchased from Abcam (Cambridge, UK), and total Erk1/2, Akt, and P38MAPK were from BD Biosciences (USA). An antibody against NRP1 was purchased from R&D Systems (Minneapolis, MN, USA), and HRP (horseradish peroxidase)-conjugated goat anti-mouse IgG and HRP-conjugated goat anti-rabbit IgG were purchased from Santa Cruz Biotechnology.

### Immunoblotting

Total protein was extracted from the transfected cells using RIPA lysis buffer (Beyotime, China) according to the manufacturer's instructions. After the whole-cell protein extracts were quantified using the BCA protein assay, equivalent amounts of cell lysates were resolved by 10% SDS polyacrylamide gel electrophoresis and were transferred onto a polyvinylidene fluoride membrane, which was then blocked in 5% non-fat milk in TBST for 1 hour at 4°C. The blots were then incubated with primary antibodies. After incubation with HRP-conjugated secondary antibodies, the protein bands were visualized using an enhanced chemiluminescence reagent (Millipore, Billerica, MA, USA). The following antibody dilutions were used: anti-fibronectin, 1∶200; anti-vimentin, anti-E-cadherin and anti-N-cadherin, 1∶1200; anti-SNAIL and anti-GAPDH, 1∶500; anti-phospho-Erk1/2, anti-phospho-Akt, and anti-phospho-P38MAPK, 1∶2000; anti-NRP1, 1∶600; anti-total Erk1/2, anti-Akt, and anti-P38MAPK, 1∶500; and HRP-conjugated IgG, 1∶7000.

### Xenograft Experiments

Male athymic nude mice 6 to 8 weeks of age were obtained from the Animal Experimental Center of Chongqing Medical University and were acclimated for 2 weeks. This study was conducted in strict accordance with the recommendations of the Guide for the Care and Use of Laboratory Animals of Chongqing Medical University. The protocol was approved by the Committee on the Ethics of Animal Experiments of Chongqing Medical University. All surgeries were performed under sodium pentobarbital anesthesia, and all efforts were made to minimize suffering. Equal numbers of AGS cells (10^6^) with forced expression of miR-338 or cont-miR, with or without NRP1 restoration, were suspended in 100 µl PBS and injected subcutaneously into the right rear flank of each mouse (10 mice per group).Tumor growth was monitored daily in each group. The tumor volume was calculated using the formula V = 1/2 a×b2, where a is the longest tumor axis, and b is the shortest tumor axis. The mice were sacrificed 5 weeks later and the tumors were divided into two parts. One part was fixed in formol for subsequent immunohistochemical analysis, and the other part was preserved in liquidnitrogen for western blotting or qRT-PCR.

### Immunohistochemistry

Tumors preserved in formalin were placed in paraffin blocks and sectioned onto positively charged microscope slides. The sections were deparaffinized in xylene, hydrated in graded alcohol, and pretreated for antigen retrieval in citrate buffer for 20 min in a 98°C steamer (CD34 and NRP1). The sections were incubated at 4°C overnight with CD34 (1∶200, Santa Cruz Biotechnology) and NRP1 (1∶100 R&D Systems). Immunostaining was performed using the UltraSensitive S-P Detection Kit (KIT-9720, Maixin, Fuzhou, China), and the color was developed using a DAB kit (PW017, Sangon Biotech, Shanghai, China).Subsequently, the sections were counterstained with hematoxylin. The slides were then stained with H&E to assess morphology.

### Quantification of Immunohistochemical Stain Intensity

To determine the tumor microvessel density, five randomly selected brightfield microscopic images (magnification 40×; area 0.89 mm^2^) per sample were obtained as described above, and the positively stained microvessels were counted using the ImageJ program. The red channel, corresponding to CD34 staining, was isolated and digitized into a binary image, with black indicating stained vessels and white indicating no staining. Vessels with lumens were digitally filled, and a composite D-MVA was quantitated. Using the ImageJ program, brown-colored images specific for DAB staining were extracted by a color deconvolution macro. All the intensity values within the same group were averaged to calculate the ratios between different groups.

### Statistical Analysis

SPSS 17.0 software was used for the statistical analysis. The data are presented as the means±standard deviation (s.d.). Group comparisons were performed using Student's t-test. Differences were considered significant at p<0.05.

## Results

### MIR Selection

To be included in our subsequent analysis, protein targets of NRP1 had to be concordantly predicted by at least three of the following four prediction tools: Miranda (http://www.microrna.org), PicTar (http://pictar.bio.nyu.edu/), TargetScan (http://genes.mit.edu/targetscan/index.html) and miRDB (mirdb.org/miRDB/). Based on the January 2012 release version, we found 11 microRNAs that potentially targeted NRP1. We had previously identified several miRNAs abnormally expressed in gastric carcinoma using a gene chip[19] (Table 1). Based on the above two,we predicted an upstream miRNA of NRP1: miR-338.

**Table 1:** miRNAs differentially expressed in gastric cancer samples compared with adjacent normal samples.

Down-regulated(n = 19)	Up-regulated(n = 7)
miR-9	miR-518b
miR-490	miR-26b
miR-188	miR-212
miR-503	miR-320
miR-545	miR-409-3p
miR-575	miR-30a-5p
miR-649	miR-379
miR-338	
miR-652	
miR-370	
miR-433	
miR-155	
miR-630	
miR-611	
miR-567	
miR-197	
miR-19b	
miR-353	
miR-551a	

### miR-338 Is Down-Regulated in Gastric Cancer Tissues and Cell Lines

**Figure 1:**
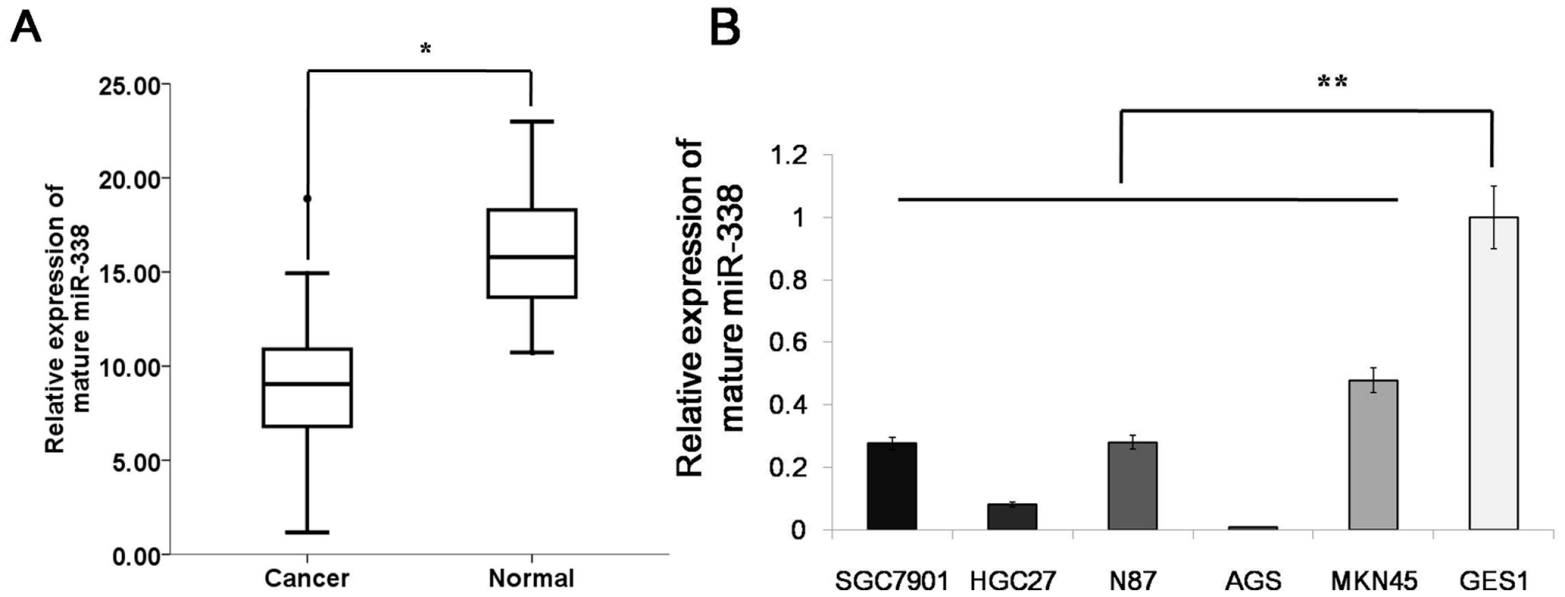
miR-338 is down-regulated in gastric cancer tissues and cell lines. (A) The expression levels of mature miR-338 in gastric cancer (n = 41) and adjacent normal mucosa tissues (n = 24) were determined using quantitative PCR. Both the gastric cancer and adjacent normal mucosa samples were fresh dissected. The expression level of mature miR-338 in the gastric cancer tissues was significantly lower than in the adjacent normal mucosa tissues. The data from the cell lines and the human samples are shown separately. * p<0.01(B)miR-338 was decreased in the gastric cancer cell lines (SGC7901, HGC27, AGS, MKN45 and N87) compared with the normal gastric mucosa cell line (GES1). The data represent the means± s.d. (n = 3) of the cell lines. ** p<0.01.

To explore the roles of miR-338 in human gastric cancer development, we measured its levels of expression in 41 human gastric cancer samples and 24 adjacent normal mucosa samples. According to a qRT-PCR analysis, the level of miR-338 expression was significantly reduced in the tumor tissues compared with the adjacent normal mucosa tissues (Fig. 1A). In addition, miR-338 expression was decreased in the gastric cancer cell lines (SGC7901, HGC27, AGS, MKN45 and N87) compared with the normal gastric mucosa cell line (GES1) (Fig 1B). These results suggest that miR-338 is down-regulated in gastric cancer cells, a finding that might be relevant to human gastric cancer development.

### miR-338 Directly Targets Oncogenic NRP1

**Figure 2:**
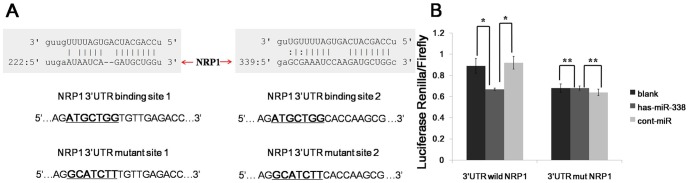
miR-338 decreases NRP1 expression by directly targeting its 3′UTR. (A) Luciferase reporter plasmids were constructed by the insertion of the full-length NRP1 3′UTR into a region immediately downstream of the luciferase gene. The sequences of two predicted miR-338 binding sites within the NRP1 3′UTR, including thefull-length wild-type UTR and a mutant binding site, are shown. (B) The relative luciferase activity was analyzed after the above reporter plasmids or a control reporter plasmid were co-transfected with miR-338 mimics or control mimics into MKN45 cells. The data represent the means±s.d.; * p<0.01, ** p>0.05.

NRP1 has been reported to be an important molecule that drives gastric cancer migration and invasion. Using prediction tools, we predicted that the target miRNA of NRP1 was miR-338. To further confirm that miR-338 directly targets oncogene NRP1, we performed luciferase reporter assays to examine whether miR-338 interacts directly with its target oncogenic NRP1. We identified two potential binding sites for miR-338 at the 3′UTR of the NRP1 mRNA. To determine whether NRP1 is regulated by miR-338 through direct binding to the 3′UTR of NRP1, we constructed a series of 3′UTR fragments, including the full-length wild-type NRP1 3′UTR, a binding site 1 mutant and a binding site 2 mutant (Fig. 2A). These fragments were then inserted into the psiCHECK2 luciferase reporter plasmid. We found that the co-transfection of miR-338 and the wild-type NRP1 3′UTR caused a significant decrease in luciferase units compared with the controls. However, the co-transfection of miR-338 and the mutant NRP13′UTR did not cause a decrease in luciferase units (Fig. 2B). These results suggest that miR-338 targets NRP1 directly.

### miR-338 Inhibits Gastric Cancer Cell Migration, Invasion and Proliferation and Promotes Apoptosis by NRP1

**Figure 3:**
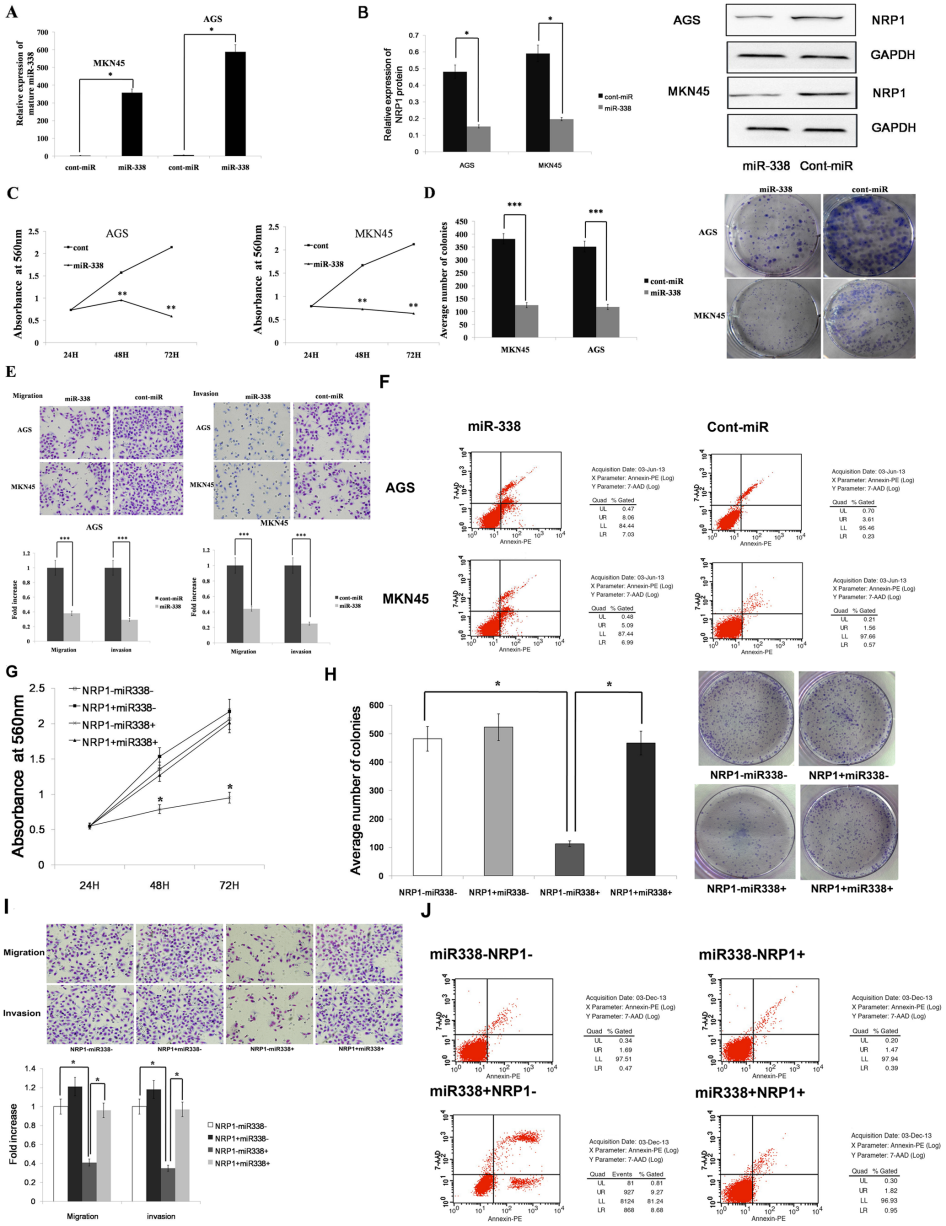
miR-338 inhibits gastric cancer cell migration, invasion and proliferation, and it promotes apoptosis. (A) The miR-338 expression was significantly increased in gastric cancer cells after infection with LV-hsa-mir-338. (B) The NRP1 expression was significantly decreased in gastric cancer cells after infection with LV-hsa-mir-338. (C) Gastric cancer cell proliferation was significantly reduced after LV-hsa-mir-338 infection compared with cont-miR infection. (D) miR-338 overexpression significantly inhibited the colony-forming ability of gastric cancer cells. (E) miR-338 overexpression significantly reduced the migration and invasion capacities of AGS and MKN45 cells compared with the controls. (F) miR-338 overexpression significantly increased gastric cancer cell apoptosis. (G-J) Gastric cancer cell migration, invasion,proliferation and apoptosis are restored after NRP1 restoration. The data represent the means±s.d.; * p<0.001, ** p<0.05, *** p<0.01.

To examine the functional significance of miR-338 overexpression in gastric cancer, we infected the gastric cancer cell lines AGS and MKN45 with LV-hsa-mir-338. Compared with the expression of cont-miR, miR-338 was significantly overexpressed in the AGS and MKN45 cell lines after infection with LV-hsa-mir-338 (Figure 3A). We also found that, compared with cont-miR, the expression of NRP1 was significantly down-regulated in the AGS and MKN45 cell lines after infection with LV-hsa-mir-338 (Figure 3B). The cells with forced expression of miR-338 exhibited significantly decreased proliferation compared with the cells with forced expression of cont-miR (Figure 3C). The miR-338-infected cells also exhibited reduced colony formation ability: the number of foci in the miR-338-expressing cells was decreased compared with the cont-miR-infected cells (Figure 3D). The transwell migration and Matrigel invasion assays demonstrated that miR-338 significantly reduced the migration and invasion capacities of the AGS and MKN45 cells (Figure 3E). The fluorescence-activated cell sorting (FACS) analysis showed that the forced expression of miR-338 lead to gastric cancer cell apoptosis. The percentage of total apoptotic cells (early apoptotic + late apoptotic) significantly increased by 11% in response to miR-338 overexpression compared with cont-miR overexpression in AGS cells. A 10% increase in the number of apoptotic cells was observed in the MKN45 cells with miR-338 overexpression compared to cont-miR (Figure 3F). miR-338 could regulate the expression of NRP1 by directly inhibiting NRP1 transcript or other indirect circuits, so we next ascertained whether the reduction of NRP1 expression could explain the inhibition of gastric cancer cell migration, invasion and proliferation observed after the forced expression of miR-338. We therefore forced the expression of miR-338 in AGS cells together with a construct containing the NRP1 coding sequence but lacking the 3′UTR of the NRP1 mRNA. As a result, this construct yielded an NRP1 mRNA that was resistant to miR-338. We found that gastric cancer cell migration, invasion, proliferation and apoptosis were restored in the AGS cell line with forced miR-338 expression and NRP1 restoration (Figure 3G–3J). Those results show that miR-338 inhibits gastric cancer cell migration, invasion and proliferation and promotes apoptosis by targeting NRP1.

### Effect of miR-338 on Signaling Pathways in AGS Cells

**Figure 4:**
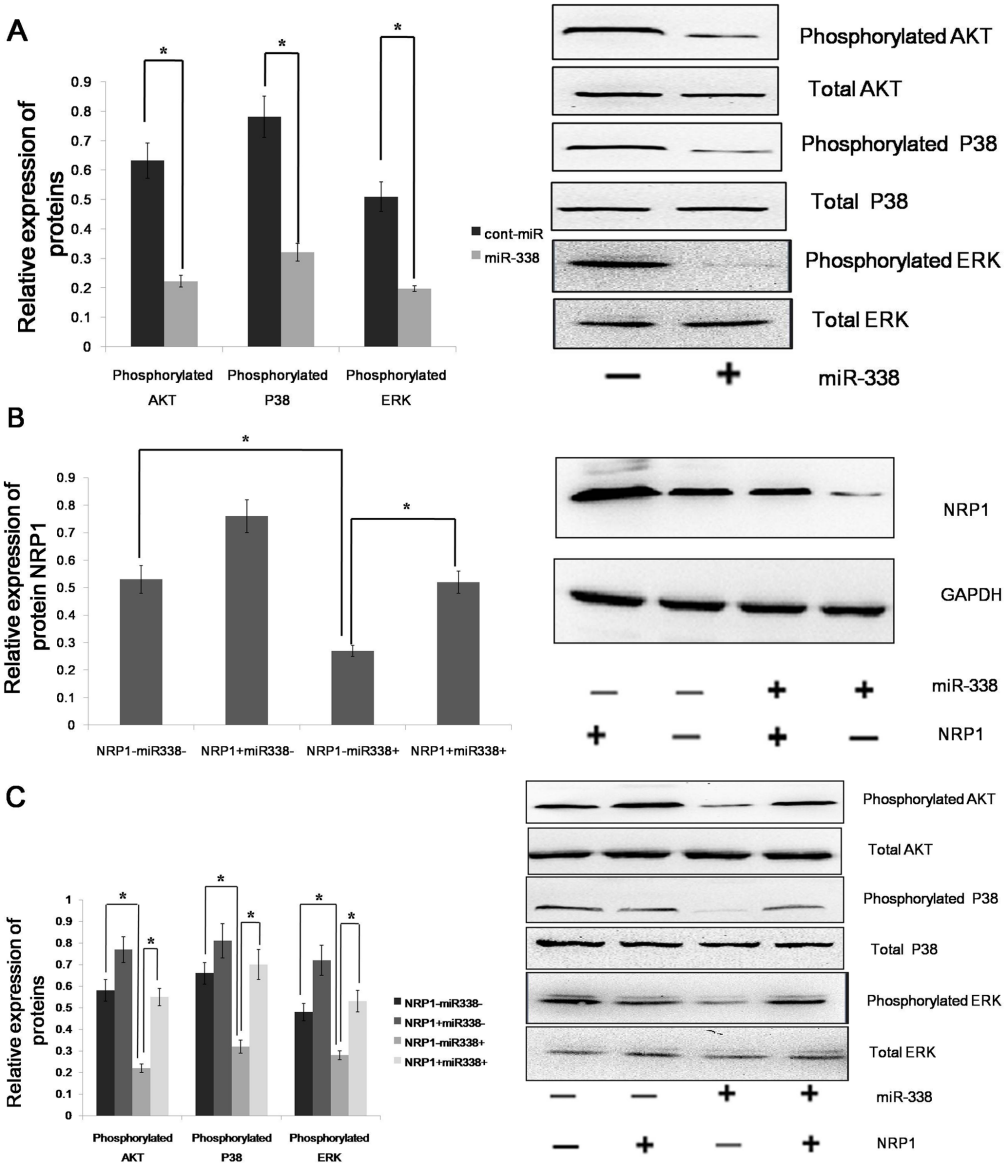
miR-338 up-regulates the phosphorylation of ERK1/2, P38 MAPK and Akt via NRP1. (A)The phosphorylation and total expression levels of ERK1/2, Akt and P38MAPK in AGS cells infected with LV-hsa-mir-338 or cont-miR. (B) An immunoblot analysis of NRP1 expression in AGS cells infected with LV-hsa-mir-338 or cont-miR, with or without NRP1 restoration. (C) The phosphorylation and total expression levels of ERK1/2, Akt and P38MAPK in AGS cells infected with LV-hsa-mir-338 or cont-miR, with or without NRP1 restoration. The expression levels of the phosphorylated proteins were normalized to those of the respective total proteins. The data represent the means±s.d.; *p<0.01.

As a non-tyrosine-kinase receptor, NRP1 can moderately increase the expression of the phosphorylation of Erk1/2, Akt, and P38MAPK to promote tumor cell proliferation and metastasis, as well as to curb the apoptosis and immune responses. Because NRP1 is a downstream target of miR-338, we assumed that miR-338 could decrease the expression of the phosphorylation of Erk1/2, Akt, and P38MAPK. We found that the forced expression of miR-338 inhibited the phosphorylation of Erk1/2, but the relative expression level of total Erk1/2 was not significantly altered. miR-338 could also decrease the phosphorylation of P38 MAPK and Akt(Fig 4A). miR-338 could bind the 3′UTR of the NRP1 mRNA to regulate the phosphorylation of ERK1/2, Akt and P38MAPK; however, we did not know whether miR-338 could bind the 3′UTRs of other genes to achieve an equal effect. So rescue experiment was performed, and the restoration of NRP1 expression was confirmed through an immunoblot analysis (Fig 4B). We found that the phosphorylation levels of Erk1/2, Akt and P38MAPK were not significantly altered in the AGS cells with forced miR-338 expression and NRP1 restoration (Fig 4C). Therefore, we conclude that miR-338 regulates the phosphorylation of ERK1/2, P38 MAPK and Akt via NRP1.

### miR-338 Determines the Epithelial Phenotype of Gastric Cancer

**Figure 5:**
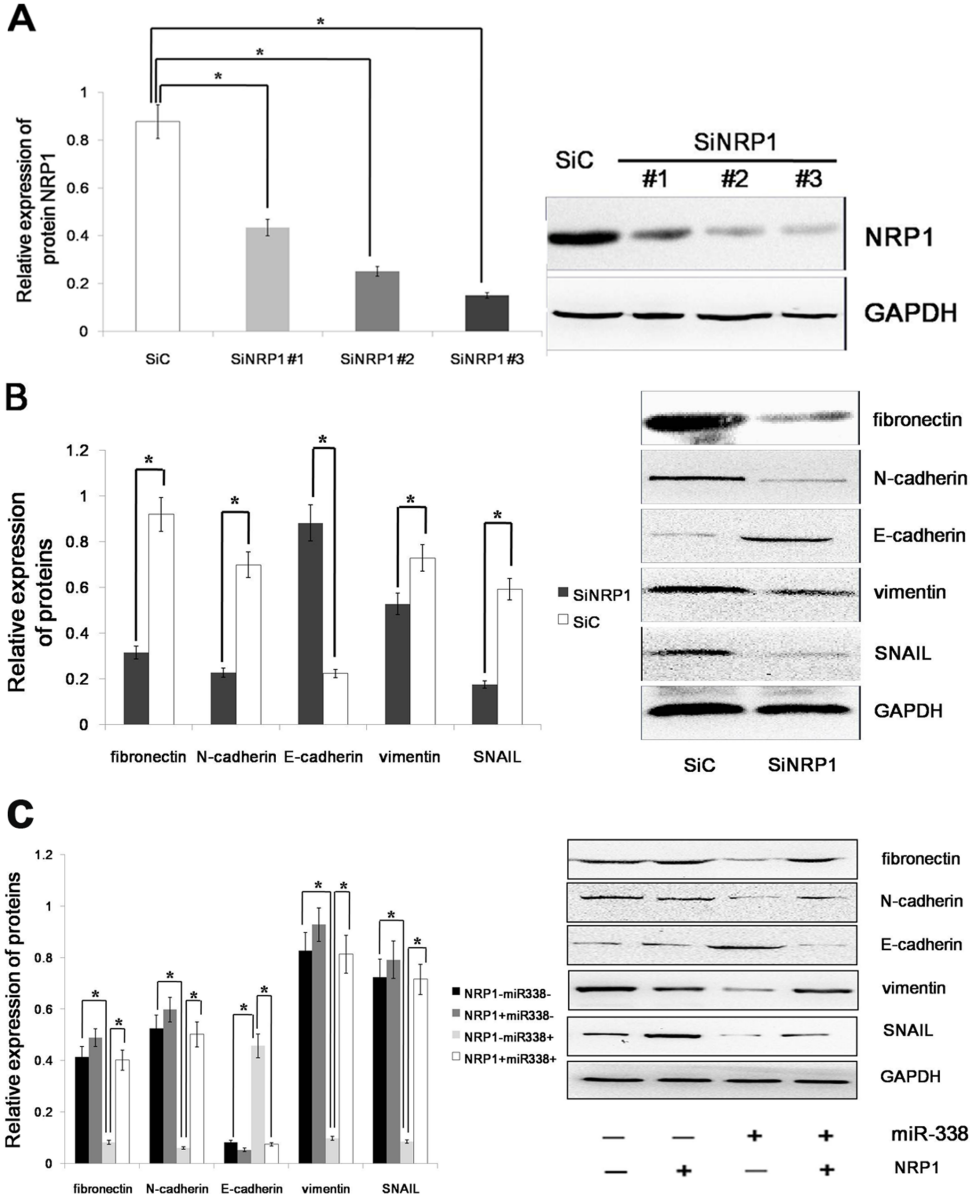
miR-338 promotes an epithelial phenotype in gastric cancer. (A) Right panel: NRP1 expression was detected by western blot in AGS cells after treatment with 3 independent siRNA sequences (siNRP1) or a control (siC). Left panel: Relative expression of NRP1 was shown in the histogram. (B) Right panel: An immunoblot analysis of N-cadherin, vimentin, fibronectin, E-cadherin and SNAIL in AGS cells transfected with siNRP1 or siC. Left panel: Relative expression of proteins was shown in the histogram. (C) An immunoblot analysis of N-cadherin, vimentin, fibronectin, E-cadherin and SNAIL in AGS cells infected with LV-hsa-mir-338 or cont-miR, with or without NRP1 restoration. The protein expression levels were normalized to GAPDH. The data represent the means±s.d.; * p<0.01.

EMT is an important mechanism associated with cancer invasiveness and metastasis. The phenomenon of EMT is defined as the transition of epithelial cells to fibroblastoid- or mesenchymal-like cells. EMT is characterized by the loss of epithelial markers and the acquisition of mesenchymal components. E-cadherin, occludin and cytokeratin are downregulated during EMT, whereas N-cadherin, vimentin, fibronectin and SNAIL are upregulated. NRP1 enhances signaling via three major pathways that have been linked to EMT, i.e., TGF-β, Hh and HGF/cMet. To find the role of NRP1 in sustaining the mesenchymal phenotype of gastric cells, we knocked down the expression of NRP1 by RNA interference (RNAi) (Fig. 5A) and examined the expression of mesenchymal markers such as fibronectin, vimentin, N-cadherin and SNAIL, as well as the epithelial marker E-cadherin, in AGS cells. We found that the expression levels of fibronectin, vimentin, N-cadherin and SNAIL were decreased but the expression of E-cadherin was increased in the NRP1-depleted tumor cells (Fig. 5B). The result shows that NRP1 can drive EMT process in gastric cancer. Because NRP1 is a downstream target of miR-338, we assumed that miR-338 could determine the epithelial phenotype of gastric cancer. To determine whether the molecular changes typical of a reduced EMT occurred in miR-338-expressing cells, we examined the expression of mesenchymal and epithelial markers in AGS cells. The immunoblot analysis showed that the expression levels of fibronectin, vimentin, N-cadherin and SNAIL were decreased in the AGS cells with the forced expression of miR-338. Furthermore, the forced expression of miR-338 increased the expression of E-cadherin in the AGS cell line, whereas the control-infected cells remained E-cadherin negative. We found that miR-338 regulated the phosphorylation of ERK1/2, P38 MAPK and Akt via NRP1; therefore, it was necessary to ascertain whether miR-338 could regulate EMT via NRP1. The immunoblot analysis showed that the expression of the above mesenchymal markers in the miR-338-expressing cells was restored to the normal level by the restoration of NRP1 expression (Fig. 5C). Altogether, these results demonstrated that miR-338 could inhibit EMT via NRP1 in gastric cancer cells.

### miR-338 Decreases Tumor Growth and Suppresses D-MVA by Targeting NRP1 *In Vivo*

**Figure 6:**
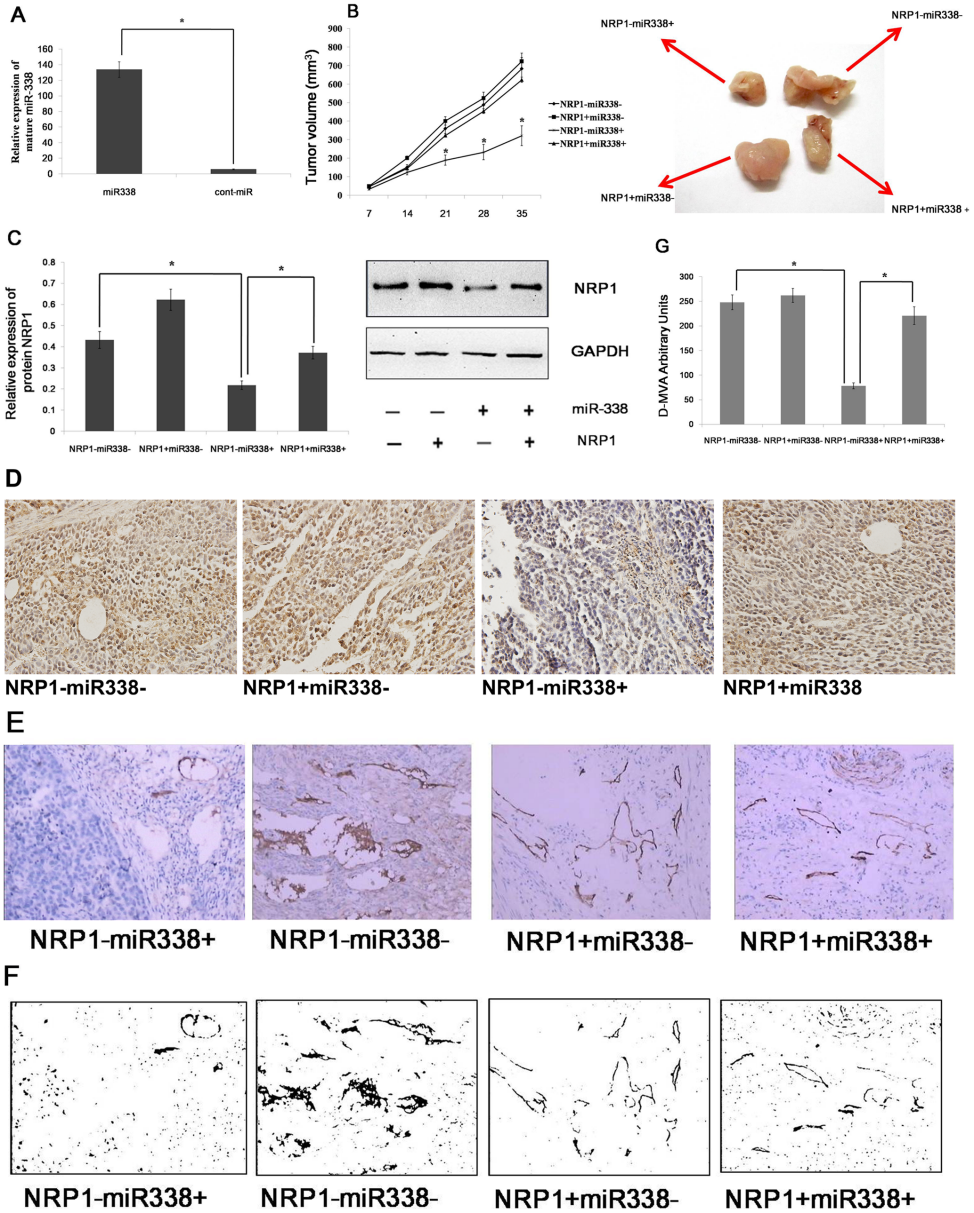
miR-338 decreases tumor growth and suppresses D-MVA by targeting NRP1 in vivo. (A) miR-338 expression in nude mouse tumors was measured using qRT-PCR. (B) Left panel: primary tumor growth after the orthotopic injection of 1×10^6^ AGS cells with the forced expression of miR-338 or cont-miR, with or without NRP1 restoration. Right panel: a representative image of xenograft tumors from nude mice. (C) NRP1 expression in nude mouse tumors was detected by western blot. (D) NRP1 expression in nude mouse tumors was detected by immunohistochemical staining. The arrows indicate NRP1. (E) CD34staining revealed few patent vessels per field in the tumors that overexpressed miR-338; however, the vessel number was restored in the tumors that overexpressed NRP1. (F) Binary images of CD34 staining with lumens digitally filled show lower D-MVA in miR-338 tumor sections and D-MVA was restored in the tumors that overexpressed NRP1. (G) The D-MVA was decreased in nude mouse tumors that overexpressed miR-338; however, the D-MVA was restored in the tumors that overexpressed NRP1. The data represent the means±s.d.; * p<0.01.

Based on the observed decreases in migratory, invasive and proliferative behaviors in AGS and MKN45 cells infected with LV-hsa-mir-338, we next investigated the role of miR-338 in growth in vivo. We subcutaneously inoculated nude mice with equal numbers (1×10^6^ cells per mouse) of AGS cells with the forced expression of miR-338 or cont-miR, with or without NRP1 restoration. Tumor incidence was assessed biweekly, and tumors appeared in all the mice. miR-338 expression in the nude mouse tumors was measured using qRT-PCR, and we found that miR-338 expression significantly increased in the tumors that overexpressed miR-338 (Figure 6A). The forced expression of miR-338 significantly inhibited tumor growth in vivo, but the overexpression of NRP1 could restore tumor growth(Figure 6B). Next, we examined NRP1 expression in the nude mouse tumors by western blot and immunohistochemistry. We found that NRP1 expression significantly decreased in the tumors that overexpressed miR-338; however, NRP1 expression was restored in the tumors that overexpressed NRP1 (Figure 6C, 4D). Thus, we inferred that miR-338 inhibited tumor growth by curbing NRP1 expression in vivo. NRP1 can boost tumorangiogenesis; therefore, we hypothesized that the forced expression of miR-338 would inhibit tumorangiogenesis via NPR1. Immunohistochemical staining for CD34 was used to evaluate the number and morphologic characteristics of blood vessels within each tumor. The vessels were enumerated by counting the number of structures with discrete staining within each field without regard to vessel size or patency. The vessels in the tumors that overexpressed miR-338 were subjectively less than the cont-miR-overexpressing tumors; however, the number of the vessels was restored in the tumors that overexpressed NRP1 (Figure 6E). The digitized microvascular area (D-MVA) was analyzed to incorporate vessel size and patency into our analysis of the tumor vasculature by providing an estimate of the integrated lumen area and, presumably, the blood flow orthogonal to the tumor section. The D-MVA was reduced in the miR-338 overexpressed tumors. To our surprise, we found that NRP1-overexpressed tumors did not significantly show increased angiogenesis, but the reduction in D-MVA due to miR-338 overexpression was restored in the tumors that overexpressed NRP1 (Figure 6F and 6G). We speculate that NRP1 expression was already upregulated in gastric cancer, so further overexpressed NRP1 cannot significantly increase tumor vasculature but can restore tumor vasculature which has been reduced by miR-338. These results indicated that miR-338 reduces tumor growth and suppresses D-MVA by targeting NRP1 in vivo.

## Discussion

Using gene chips and bioinformatics, we predicted that the target miRNA of NRP1 was miR-338. Research has shown that the expression of miR-338 varies in different tumors. Huang XH et al. showed that miR-338-3p suppressed liver cancer cell invasion by targeting smoothened [20]. Moreover, in melanomas, miR-193a, miR-338, and miR-565 were shown to be underexpressed in patients with a BRAF mutation [21]. From these data, we inferred that miR-338 might act as a tumor suppressor. There is no published literature regarding whether miR-338 is underexpressed in gastric cancer. In our study, the expression levels of miR-338 were first measured in 41 human gastric cancer samples and 24 samples of adjacent normal mucosa tissues. We then evaluated the expression of miR-338 in five gastric cancer cell lines and in normal gastric mucosa cell lines. We found that the expression of miR-338 was down-regulated in both the tumor tissues and the cancer cell lines. Moreover, the overexpressed miR-338 could inhibit gastric cancer cell migration, invasion and proliferation, and promote apoptosis. Meanwhile, those tumourigenic qualities can be completely restored by NRP1 overexpression. In addition, the luciferase reporter assays suggested that miR-338 targets NRP1 directly. Thus, we conclude that miR-338 acts as a potential tumor suppressor in gastric cancer, a function that is accomplished by curbing the expression of NRP1.

As a non-tyrosine-kinase receptor, NRP1 can moderately increase the expression of the phosphorylation of Erk1/2, Akt, and P38MAPK. M Akagi et al. found that the inhibition of NRP-1 reduced the expression of the phosphorylation of Erk1/2, Akt, and P38MAPK [22]. Additionally, research has shown that the expression of ERK1/2 phosphorylation was up-regulated in NRP-1-overexpressing cells [23]. In this study, we found that overexpressed miR-338 could decrease the expression of the phosphorylation of Erk1/2, Akt, and P38MAPK, an effect that was reversed upon NRP1 restoration. The activation of ERK, Akt and P38MAPK in association with apoptosis resistance/cell survival has been well documented in a variety of model systems [24], [25], [26]. Thus, we conclude that miR-338 inhibits gastric cancer cell migration, invasion and proliferation, as well as promoting apoptosis, by decreasing the expression of NRP1, which increases ERK, Akt and P38MAPK signaling.

The phenotypic transition from an epithelial to a mesenchymal-like cell state represents an important mechanism of epithelial plasticity and cancer metastasis. MicroRNAs have recently emerged as potent regulators of EMT due to their ability to target multiple components involved in epithelial integrity or mesenchymal traits. The miR-200 family has been shown to directly target EMT transcription factor families [27]. In human mammary epithelial cells, miR-9 directly targets E-cadherin, thus promoting the mesenchymal phenotype, including increased cell migration and invasion [28]. MiR-27 promotes human gastric cancer cell metastasis by inducing the epithelial-to-mesenchymal transition[29]. Nrp1 enhances signaling in three major pathways that have been linked to EMT, i.e., TGF-β, Hh and HGF/cMet. TGF-β plays a major role in EMT by regulating the expression of multiple genes and pathways, as recently reviewed by Fuxe et al.[30]. In the present study, we found that in gastric cancer, overexpressed NRP1 promoted EMT and siNRP1 restrained EMT, whereas the forced expression of miR-338 inhibited EMT. miR-338 may therefore regulate gastric cancer cell EMT via NRP1.

In vivo, we found that tumor growth was significantly inhibited by the forced expression of miR-338 but was restored by NRP1 overexpression. Thus, we can infer that the reduction in tumor growth may have been due to the decrease in NRP1, which was curbed by miR-338. Wu et al found that silenced NRP1 on oral cancer cells can regulate xenografted tumor angiogenesis [31]. Meanwhile, Dallas et al found that reduced NRP-2 expression on pancreatic tumor cells can attenuate the tumor D-MVA in vivo [32]. These findings indicate that reduced NRP expression in tumor cells can inhibit xenografted tumor angiogenesis. In our study, NRP1 expression was reduced by miR-338 in tumor cells and reduced NRP1 expression attenuate the xenografted tumor D-MVA. Moreover, the reduced tumor D-MVA could be restored in the tumors that overexpressed NRP1. These data suggest that miR-338 can attenuate tumor D-MVA via NRP1. But why reduced NRP1 in gastric cancer cells can curb the D-MVA of xenografted tumor? We guess that the effects on development of the tumor vasculature may be due to altered angiogenic mediator expression in the tumor cells themselves, and we will detect protein levels of several known angiogenic mediators such as VEGF-A, VEGF-C and so on in our next study. In general, NRP1 may mediate the effects of miR-338 on tumor progression indirectly by affecting angiogenesis, or directly, through its effects on tumor cells.

In conclusion, our study is the first to document the tumor suppressor role of miR-338 in gastric cancer. miR-338 can decrease migratory, invasive and proliferative behaviors by attenuating the expression of NRP1. Our findings indicate that the restoration of the tumor suppressor miR-338 might be useful in the treatment of gastric cancer.
